# Unraveling the Effects of a Talimogene Laherparepvec (T-VEC)-Induced Tumor Oncolysate on Myeloid Dendritic Cells

**DOI:** 10.3389/fimmu.2021.733506

**Published:** 2021-10-28

**Authors:** Jens Tijtgat, Jolien De Munck, Inès Dufait, Julia Katharina Schwarze, Ivan Van Riet, Lorenzo Franceschini, Karine Breckpot, Joeri L. Aerts, Bart Neyns, Sandra Tuyaerts

**Affiliations:** ^1^ Department of Medical Oncology/Laboratory of Medical and Molecular Oncology (LMMO), Vrije Universiteit Brussel (VUB), Universitair Ziekenhuis Brussel (UZ Brussel), Brussels, Belgium; ^2^ Neuro-Aging and Viro-Immunotherapy (NAVI) Research Group, Vrije Universiteit Brussel (VUB), Brussels, Belgium; ^3^ Department of Radiotherapy/Laboratory of Translational Radiation Oncology, Supportive Care and Physics (TROP), Universitair Ziekenhuis Brussel (UZ Brussel)/Vrije Universiteit Brussel (VUB), Brussels, Belgium; ^4^ Stem Cell Laboratory, Department of Hematology, Vrije Universiteit Brussel (VUB), Universitair Ziekenhuis Brussel (UZ Brussel), Brussels, Belgium; ^5^ Laboratory for Molecular and Cellular Therapy (LMCT), Department of Biomedical Sciences, Vrije Universiteit Brussel (VUB), Brussels, Belgium

**Keywords:** melanoma, myeloid dendritic cell, BDCA-1, BDCA-3, Talimogene laherparepvec, cell therapy, immunotherapy

## Abstract

T-VEC, a HSV-1 derived oncolytic virus, is approved for the treatment of advanced melanoma. The mechanisms that underly the systemic anti-tumor effect that is seen following intratumoral injection have not yet been studied but are likely to be mediated by myeloid dendritic cells (myDC) that initiate an adaptive immune response. In this study we could demonstrate that T-VEC is non-toxic for human myDC. T-VEC and a T-VEC oncolysate of melanoma cell lines were able to mature human myDC. myDC were able to take up lysed melanoma cells and cross-present melanoma-derived tumor antigens to antigen-specific T cells. Our results support the possible role of myDC as mediators of an adaptive anti-tumor effect and intratumoral co-administration of T-VEC plus autologous myDC could be a complementary treatment option. A clinical trial that investigates this hypothesis is currently ongoing.

## Introduction

Dendritic cells (DC) are essential for the initiation of an adaptive immune response. They act as a bridge between the innate and the adaptive immune system using their unique capabilities to activate naive lymphocytes by capturing, processing and presenting antigens. DC are generally characterized by a high expression of major histocompatibility complex (MHC) class II molecules and CD11c, but are extremely heterogeneous both in phenotype and function. All human DC arise from a CD34^+^ hematopoietic precursor, and differentiate subsequently into monocyte, macrophage and DC precursor cells (MDP), common DC precursor cells (CDP) and then into either plasmacytoid DC (pDC) or preclassical DC (pre-cDC). Pre-cDC can evolve into two types of classical or conventional DC (cDC), also called myeloid DC (myDC): cDC1 and cDC2. cDC1 are characterized by expression of CD141/BDCA-3, XCR1, CLEC9A and the BATF3 transcription factor. cDC2 are more heterogenous. They are characterized by expression of CD1c, BDCA-1 and SIRPα and are the most abundant type of cDC in the blood circulation (1). Recently, this classification was refined based on single cell RNA sequencing data that identified in total six different types of DC. Within this novel classification, cDC1 correspond to the DC1 subclass. cDC2 are subclassified in DC2 (MHC class II-like) and DC3 (CD14^+^ monocyte-like). Both cell types arise from a specific circulating and dividing cDC progenitor cell (2). For therapeutic use, another type of DC is frequently used: monocyte-derived DC (moDC). These are generated *in vitro* by stimulating CD34^+^ precursors or CD14^+^ monocytes with GM-CSF and TNF-alpha (CD34^+^) or IL-4 (monocytes). Not much is known about the *in vivo* differentiation of monocytes into moDC (3).

However, recent observations indicate that moDC might not be the best suited DC type to use for therapeutic purposes. It has been demonstrated that moDC have a decreased migratory capacity, present with a more exhausted phenotype (decreased cytokine secretion and T cell stimulatory capacity) and are generally less potent. Our research focuses on cDC1 and cDC2 as these cell types have been shown to be able to re-invigorate the cancer immunity cycle and are key to the cross-presentation of tumor antigens (4, 5). It has been shown that these cells are deficient in several cancer types, and deficiency of these cell types is correlated with a worse outcome (6). However, it has long been impossible to isolate these cells in sufficient numbers to allow therapeutic use, due to the absence of antibodies of sufficient quality (affinity and specificity) to perform their isolation. cDC1 (CD141^+^) are only present in very limited quantities in the blood (one-tenth the frequency of cDC2) (7). cDC1 have been found to be of critical importance in ‘relicensing’ the anti-tumor activity of CD8^+^ cytotoxic T cells in the tumor micro-environment. Recently, it has been shown that cDC1 are important for early priming of CD4^+^ helper T cells even though this was long hypothesized to be the specialization of cDC2 (8–10). Only limited (clinical) data is currently available regarding the therapeutic use of these cell types (11).

DC need to successfully perform several functions to trigger an effective adaptive immune response. First, they should take up tumor-associated antigens, then (concurrently) they need to be stimulated in order to become activated. After activation, mature DC must migrate to the lymph node area where they present tumor antigens to antigen-specific T cells. Adequate stimulation must be present in the tumor environment for DC to become mature. Upon induction of cell death, cells release a variety of molecules in their environment depending on both cell type and type of cell death. These cell death-induced mediators can be detected by various pattern recognition receptors (PRR). The most widely known PRR are the so-called Toll-like receptors (TLR) which are differentially expressed on the different types of myDC. It has been shown that CD1c^+^ myDC express all TLR except for TLR9. In contrast, CD141^+^ myDC exhibit a restricted pattern of TLR expression with high expression of TLR3 and TLR10, intermediate expression of TLR1, -2, -6 and -8, and no expression of TLR4, -5, -7 and -9. TLR9 expression is mainly restricted to pDC. However, despite the absence of TLR9 receptors, CD141^+^ cells have nevertheless shown to produce IL-12 in response to TLR9 agonists pointing to a yet unidentified receptor type (12).

Talimogene laherparepvec (T-VEC, Imlygic^®^, Amgen) is a herpes simplex virus-1 (HSV-1) derived oncolytic virus (OV) that has been approved by the Food and Drug Administration (FDA) in 2015 for the treatment of local and locally advanced cutaneous melanoma (13, 14). T-VEC selectively replicates in tumor cells and improves the immune response by inducing granulocyte-macrophage colony stimulating factor (GM-CSF) secretion by infected cells. In addition, it has been shown that T-VEC induces immunogenic cell death in melanoma cell lines with the associated release of damage-associated molecular patterns (DAMPs), as measured by release of high mobility group box-1 (HMGB-1), adenosine triphosphate (ATP) and ecto-calreticulin (CRT). HMGB-1 release results in stimulation of TLR2, TLR4 and RAGE receptors (15, 16). Release of ATP interacts with DC through the P2X7 and P2Y2 receptor in order to attract immune cells and act as a ‘find-me’ signal (17). CRT functions as an ‘eat-me’ signal by activating the CD91 (LRP1) receptor and strengthens the immune response by releasing pro-inflammatory cytokines leading to Th17 priming (18).

The mechanisms by which intratumorally injected T-VEC generates a protective systemic anti-tumor effect have not been elucidated. Presumably intratumoral T-VEC administration reactivates a cancer immunity cycle by lysing tumor cells and providing viral elements that activate antigen presenting cells. These antigen presenting cells can re-initiate an adaptive immune response. In this study we have taken advantage of the availability of human myDC obtained within the context of a clinical trial to study their interaction with T-VEC.

## Materials and Methods

### Cell lines and Reagents

The human-derived melanoma cell lines 624-mel and 938-mel were a kind gift from prof. S. Topalian (Institute for Cancer Immunotherapy, Johns Hopkins University School of Medicine) to prof. Aerts. Cells were cultured in RPMI-1640 (Life Technologies) supplemented with 10% FBS (Fetal Bovine Serum, Biochrome), 2 mM L-glutamine (Life Technologies), 100 U/mL penicillin (Life Technologies) and 100 μg/mL streptomycin (Life Technologies) at 37°C, 5% CO_2_.

Talimogene laherparepvec (T-VEC, Amgen) was stored at -80°C and thawed for use when applicable.

mRNA encoding for the α and β chain respectively of the NY-ESO-1 and gp100 T cell receptor (TCR) was produced by Prof. Breckpot’s group at the Laboratory of Molecular and Cellular Therapy (LMCT, VUB). Briefly, gBlocks^™^ (Integrated DNA Technologies, IDT) encoding for the α and β chain sequence of the T cell receptor recognizing the NY-ESO-1 peptide (SLLMWITQV) and gp100 peptide (YLEPGPVTA) were cloned into the *in-house* developed plasmid LMCT (pLMCT). pLMCT was linearized (NcoI/XhoI, Thermo Fisher Scientific) and each TCR chain sequence was cloned into the background vector *via* Gibson assembly reaction (New England Bio Labs, NEB). Cloned plasmids were screened *via* enzymatic restriction digestion and sequences were verified (Eurofins Genomics). Plasmids were prepared and purified according to Qiagen protocol (Qiagen-Plasmid Midi Prep^®^, Qiagen/Filter service). Purified TCR plasmids were linearized with BfuA1 (NEB) restriction enzyme prior mRNA *in vitro* transcription. T7 RNA polymerase (Thermo Fisher Scientific) together with co-transcriptional capping reagent CleanCap^®^ AG (TriLink Biotechnologies) were used in the iVT reaction mix. The resulting mRNA was purified *via* NaCl/EtOH precipitation and resuspended in water for injection at 1μg/μL final concentration. mRNA integrity and identity were verified with Agilent Bioanalyzer RNA 6000 Nano Kit^®^.

The NY-ESO-1 peptide (SLLMWITQV) was purchased from Fisher Scientific. The gp100 peptide (YLEPGPVTA) was a kind gift from prof. K. Breckpot.

### Incucyte^®^ Proliferation Assay

Tumor cells were plated in flat-bottom 96-well plates at a density of 1 x 10^4^ cells per well and left to adhere overnight at 37°C, 5% CO_2_ in the Incucyte^®^ Zoom instrument (Sartorius). The following day, T-VEC was added at the indicated multiplicity of infection (MOI). All conditions were tested in triplicate. Cell growth was monitored continuously with a 10x objective using 2h intervals. Cell proliferation was assessed by analyzing the occupied area (% confluence) over time using the Incucyte^®^ analysis software (Sartorius).

### GM-CSF Production

Tumor cells were plated in flat-bottom 96-well plates at a density of 1 x 10^4^ cells per well and left to adhere overnight at 37°C, 5% CO_2_. The following day, T-VEC was added at the indicated multiplicity of infection (MOI). All conditions were tested in triplicate. Supernatant was harvested after 24, 48 and 72h and stored at -20°C. GM-CSF content in these supernatants was assessed using an ELISA (Biolegend), according to manufacturer’s instructions. Optical density was read at 450 nm and 570 nm using an xMark™ absorbance spectrophotometer (Bio-Rad Laboratories) and GM-CSF concentrations were calculated using Microplate Manager software (Bio-Rad Laboratories).

### Myeloid Dendritic Cell Isolation

Isolated myDC were obtained from patients included in various clinical trials at UZ Brussel from whom excess myDC were available for translational research. Those studies have been conducted in accordance with the Declaration of Helsinki and were approved by the ethics committee of the UZ Brussel. The patients provided written informed consent to use cells that were not used for treatment for research purposes. Briefly, patients underwent a leukapheresis and next, CD14^+^ and CD19^+^ cells were depleted using CliniMACS^®^ CD14 reagent and CliniMACS^®^ CD19 reagent, followed by positive selection of CD1c (BDCA-1)^+^ and CD141 (BDCA-3)^+^ myDC using CliniMACS^®^ CD1c (BDCA-1)-biotin, CliniMACS^®^ Anti-Biotin Reagent and CliniMACS^®^ CD141 (BDCA-3) Microbeads (all Miltenyi Biotec) on the immunomagnetic CliniMACS Prodigy^®^ system (Miltenyi Biotec). Cells were cryopreserved in 7.5% DMSO – 8.25% human albumin solution and stored in the vapor phase of liquid nitrogen. The purity of the isolated BDCA-1^+^/BDCA-3^+^ cell product was analyzed by flow cytometry. 5 x 10^5^ cells were stained with CD14-FITC (Biolegend, clone HCD14), CD45-PE (Miltenyi Biotec, clone REA747), 7-AAD (Thermo Fisher Scientific), CD141-PE/Cy7 (Invitrogen, clone JAA17), CD123-APC-Vio770 (Miltenyi Biotec, clone REA918), FcER-Vioblue (Miltenyi Biotec, clone CRA1), CD11c-Alexa Fluor 700 (BD Biosciences, clone B-ly6), CD1c-Brilliant Violet 510 (BD Biosciences, clone F10/21A3), CD15-APC (Invitrogen, clone MMA) for 20 minutes at 4°C. After washing, cells were resuspended in PBS/0.5%BSA and acquired on a BD LSR Fortessa instrument (BD Biosciences). Data analysis was performed using FlowJo and FCS Express 7 software. Purity was evaluated as follows: cells were gated based on FSC/SSC characteristics and subsequently on viable 7-AAD^-^, CD45^+^ cells. On this gate, CD14 and CD15 expressing cells were excluded and then we identified CD11c^-^ CD123^+^ pDC and CD11c^+^ CD123^-^ myDC. On the myDC gate, we then identified BDCA-1^+^ DC as CD1c^+^ CD141^-^ FcεR^+^ cells and BDCA-3^+^ DC as CD1c^-^ CD141^+^ cells.

### Effect of T-VEC or Supernatant From T-VEC-Treated Melanoma Cells on myDC

Purified BDCA-1^+^/BDCA-3^+^ myDC were cultured at 1.5-2 x 10^5^ cells per 96-well (ultra-low attachment) in 200 µL X-VIVO-15 (Lonza, Belgium) supplemented with L-glutamine, penicillin-streptomycin and sodium pyruvate (DC medium) in the presence of 1000 U/mL GM-CSF (Miltenyi Biotec). T-VEC was added at a multiplicity of infection (MOI) of 1 and incubated for 24h at 37°C, 5% CO_2_. As a control, heat-inactivated T-VEC was also used (15 min at 65°C, followed by 1 min at 100°C). The combination of 20 µg/mL poly(I:C) and 4 µg/mL R848 was used as a positive control. Alternatively, supernatant of melanoma cells (624-mel or 938-mel) treated with T-VEC at MOI 1 for 24h or 48h was added to the DC and incubated for 24h at 37°C, 5% CO_2_. The supernatant of the cultures was harvested and stored at -20°C for cytokine analysis and the phenotype of the BDCA-1^+^/BDCA-3^+^ myDC was analyzed by flow cytometry.

### Flow Cytometric Analysis

For analysis of the phenotype of BDCA-1^+^/BDCA-3^+^ myDC, cells were stained with CD11c-Alexa Fluor 700 (BD Biosciences, clone B-ly6), CD1c-Brilliant Violet 510 (BD Biosciences, clone F10/21A3), CD141-PE/Cy7 (Invitrogen, clone JAA17), CD274-PE-CF594 (BD Biosciences clone MIH1), CD86-Brilliant Violet 421 (BD Biosciences, clone 2331 (FUN-1)), CD83-PE (BD Biosciences, clone HB15e), CD40-APC (BD Biosciences, clone 5C3), CD80-PE/Cy5 (BD Biosciences, clone L307.4), HLA-ABC-FITC (BD Biosciences, clone G46-2.6), Zombie Yellow (Biolegend) for 20 minutes at 4°C. After washing, cells were resuspended in PBS/0.5%BSA and acquired on a BD LSR Fortessa instrument. Data analysis was performed using FlowJo software. The gating strategy was as follows: cells were first gated on FSC/SSC characteristics, followed by gating on single cells. Next, dead cells were excluded and subsequently we gated on the CD11c^+^ population. On this gate, CD1c^+^ CD141^-^ cells were identified as BDCA-1^+^ myDC and CD1c^-^ CD141^+^ cells as BDCA-3^+^ myDC. Subsequently, we evaluated for each myDC subtype the expression of HLA-ABC, CD83, CD274/PD-L1, CD80, CD40 and CD86.

### Cytokine Detection

The supernatants of myDC treated with T-VEC or with supernatant of melanoma cells treated with T-VEC was analyzed for its content of the following cytokines: IL-10, IL-12p70, TNF-α, IFN-α-2a and IFN-λ1 using a human multiplex U-plex assay (MesoScale Diagnostics) according to manufacturer’s instructions and analyzed using a MESO QuickPlex SQ 120 instrument (MesoScale Diagnostics). Data were analyzed using the MSD Discovery Workbench software.

### Phagocytosis Assay

In order to measure the uptake of dying melanoma cells by BDCA-1^+^/BDCA-3^+^ myDC, we have set-up a co-culture assay between pHrodo™-labeled tumor cells and BDCA-1^+^/BDCA-3^+^ myDC.

To this end, 938-mel cells were plated at 2.5 x 10^5^ cells per 12-well and left to adhere overnight. The next day, T-VEC was added to these cells at an MOI of 1. After 24h incubation with T-VEC, treated cells were harvested and subsequently labeled with the pHrodo™ Deep Red Mammalian and Bacterial Cell Labeling Kit (Thermo Fisher Scientific), according to manufacturer’s instructions. Next, pHrodo™-labeled, T-VEC-treated 938-mel cells were co-cultured with BDCA-1^+^/BDCA-3^+^ myDC at different ratio’s during 2, 4 or 6h. At these time points cells were harvested and stained for CD11c-Alexa Fluor 700 (BD Biosciences, clone B-ly6), CD1c-Brilliant Violet 510 (BD Biosciences, clone F10/21A3), CD141-PE/Cy7 (Invitrogen, clone JAA17), CD45-PE (Miltenyi Biotec, clone REA747), CD123-PE/Cy7 (Miltenyi Biotec, clone REA918) and 7-AAD (Thermo Fisher Scientific) for 20 min at 4°C. After washing, cells were resuspended in PBS/0.5%BSA and acquired on a BD LSR Fortessa instrument. The pHrodo™ Deep Red dye was measured in the Cy5 channel. Data were analyzed using FlowJo software using the following gating strategy: cells were first gated based on FSC/SSC characteristics, followed by gating on single cells. Next, we selected the viable, 7-AAD^-^, CD45^+^ cells and then we selected myDC as CD11c^+^ CD123^-^ cells. On this gate, BDCA-1^+^ DC were identified as CD1c^+^ CD141^-^ cells and BDCA-3^+^ DC as CD1c^-^ CD141^+^ cells. Uptake of pHrodo™-labeled tumor cells was assessed by evaluating the percentage of Cy5-expressing cells in the CD11c^+^ DC, BDCA-1^+^ DC and BDCA-3^+^ DC gates respectively.

### Antigen Presentation Assay

On day 0, 938-mel cells were plated at 2.5 x 10^5^ cells per 12-well and left to adhere overnight. The next day, T-VEC was added to these cells at a MOI of 1. After 24h incubation with T-VEC, treated cells were harvested. On day 2, BDCA-1^+^/BDCA-3^+^ myDC from an HLA-A2-positive donor were thawed in a 37°C water bath and diluted with PBS/4% human serum. After centrifugation, cells were resuspended in DC medium and counted with trypan blue. BDCA-1^+^/BDCA-3^+^ myDC were co-cultured with T-VEC-treated 938-mel cells at different ratio’s for 24h. BDCA-1^+^/BDCA-3^+^ myDC alone were used as controls. On day 2, autologous T cells were thawed, counted with trypan blue and cultured in IMDM (Iscove’s Modified Dulbecco’s Medium, Life Technologies) containing 1% human serum, penicillin-streptomycin, L-glutamine, sodium pyruvate and non-essential amino acids (T cell medium) at a density of 5 x 10^6^ cells/mL in the presence of 250 U/mL IL-2 for 24h. On day 3, T cells were harvested, counted and electroporated with mRNA encoding the α- and β-chain of the TCR for NY-ESO-1 or gp100 (10 µg RNA per 4 x 10^6^ T cells, square wave pulse, 500V, 1 pulse, 5 ms, 4 mm cuvette). After electroporation, the cells were transferred to 2 mL T cell medium containing 20 U/mL IL-7 for 1h. Meanwhile, BDCA-1^+^/BDCA-3^+^ myDC co-cultured with T-VEC-treated 938-mel cells were harvested and counted. Next, these BDCA-1^+^/BDCA-3^+^ myDC were co-cultured overnight with the autologous TCR-electroporated T cells at a 1:1 ratio in T cell medium. Unmanipulated BDCA-1^+^/BDCA-3^+^ myDC and BDCA-1^+^/BDCA-3^+^ myDC loaded with the respective peptides (NY-ESO-1: SLLMWITQV; gp100: YLEPGPVTA), 624-mel cells (NY-ESO-1^+^, gp100^+^, HLA-A2^+^) and 938-mel cells (NY-ESO-1^+^, gp100^+^, HLA-A2^-^) were used as controls. On day 4, the supernatant of the DC-T cell co-cultures was harvested and analyzed for IFN-γ content by ELISA (Biolegend), according to manufacturer’s instructions. Optical density was read at 450 nm and 570 nm using an xMark™ absorbance spectrophotometer (Bio-Rad Laboratories) and IFNγ concentrations were calculated using Microplate Manager software (Bio-Rad Laboratories).

### Statistical Analysis

Statistical analyses were performed using the GraphPad Prism version 9.1.0 software. Live cell imaging data were analyzed using a 1-way ANOVA followed by a Friedman test for multiple comparisons. Phenotypic differences were analyzed using the Kruskal-Wallis test with Dunn’s multiple comparisons test. For the phagocytosis assay, a mixed-effects analysis was used, and the antigen presentation assay was analyzed using 1-way ANOVA.

## Results

### Cell Death Kinetics Induced by Talimogene Laherparepvec (T-VEC) on Melanoma Cell Lines

**Figure 1:**
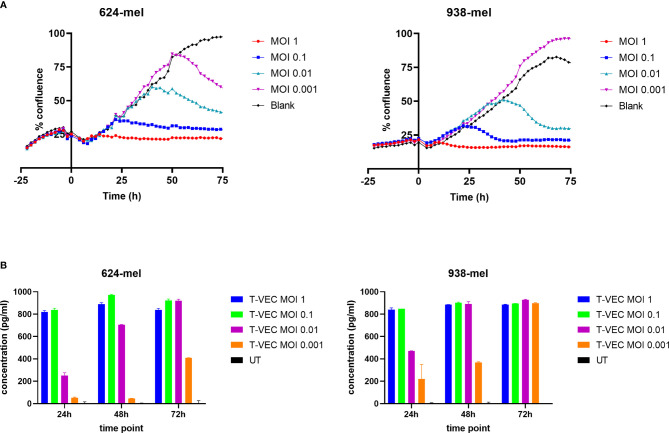
T-VEC infection of melanoma cell lines. **(A)** The confluence of cells was analyzed in real time using the confluence image mask. T-VEC was added at the indicated MOIs at time 0. The left panel shows data for the 624-mel cell line and the right panel for the 938-mel cell line. Data points depict the mean of 3 replicate wells and the graphs show 1 representative experiment out of 3. **(B)** The secretion of GM-CSF after treatment with T-VEC at the indicated time points was measured by ELISA. The left panel shows data for the 624-mel cell line and the right panel for the 938-mel cell line. Data points depict the mean ± SD of 2 replicate wells and the graphs show 1 representative experiment out of 3. UT, untreated. Statistics left panel (624-mel): At 24h, all comparisons between the different conditions were highly significant (p < 0.0001), except for UT vs T-VEC MOI 0.001 (p = 0.0123) and T-VEC MOI 1 vs T-VEC MOI 0.1 (p = 0.6944, ns). At 48h, all comparisons between the different conditions were highly significant (p < 0.0001), except for UT vs T-VEC MOI 0.001 (p = 0.0302) and T-VEC MOI 1 vs T-VEC MOI 0.1 (p = 0.0002). At 72h, all comparisons between the different conditions were highly significant (p < 0.0001), except for T-VEC MOI 1 vs T-VEC MOI 0.1 (p = 0.0002), T-VEC MOI 1 vs T-VEC MOI 0.01 (p = 0.0002) and T-VEC MOI 0.1 vs T-VEC MOI 0.01 (p > 0.9999, ns). Statistics right panel (938-mel): At 24h, all comparisons between the different conditions were highly significant (p < 0.0001), except for UT vs T-VEC MOI 0.001 (p = 0.0001) and T-VEC MOI 1 vs T-VEC MOI 0.1 (p = 0.9988, ns). At 48h, all comparisons between the different conditions were highly significant (p < 0.0001), except T-VEC MOI 1 vs T-VEC MOI 0.1 (p = 0.9913, ns), T-VEC MOI 1 vs T-VEC MOI 0.01 (p = 0.9998, ns) and T-VEC MOI 0.1 vs T-VEC MOI 0.01 (p = 0.9987, ns). At 72h, all comparisons with the UT condition were highly significant (p < 0.0001), but differences between all other conditions were non-significant.

To study the kinetics of cell death induced by T-VEC, we used the Incucyte^®^ Live Cell Imaging system to continuously monitor the cell growth upon T-VEC treatment. As illustrated in **Figure 1A**, T-VEC treatment induced a time- and dose-dependent inhibition of tumor cell growth for the melanoma cell lines 624-mel and 938-mel. The time upon which cell growth inhibition occurs is MOI-dependent. Both cell lines seem to be almost equally sensitive to T-VEC-mediated growth inhibition.

Since T-VEC encodes the gene encoding for GM-CSF, this cytokine should be produced upon successful infection. In **Figure 1B**, we show the secretion of GM-CSF by T-VEC-treated melanoma cells at 24, 48 and 72h post-infection. Also here, the amount of GM-CSF production increases over time and the levels are MOI-dependent. The differences in GM-CSF secretion between different MOI’s are statistically significant as indicated in **Figure 1B**. The levels of GM-CSF produced by both cell lines are comparable.

### T-VEC and T-VEC-Induced Oncolysate Activate Myeloid Dendritic Cells (myDC)

**Figure 2:**
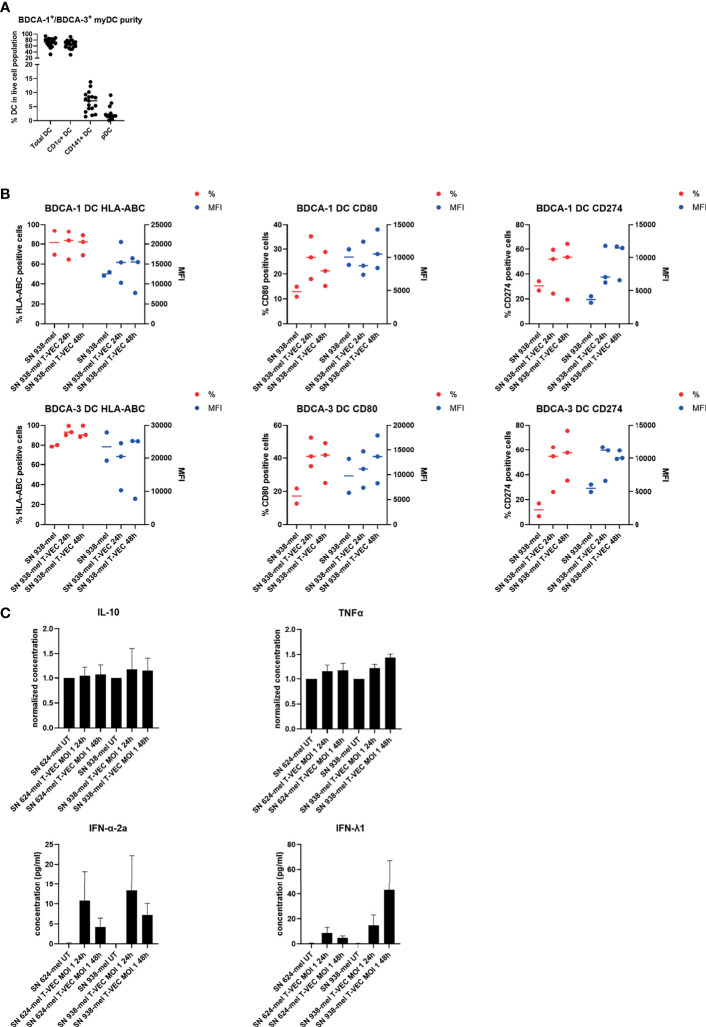
Effect of supernatant from T-VEC-treated melanoma cells on purified BDCA-1^+^/BDCA-3^+^ myDC. **(A)** The purity of the purified BDCA-1^+^/BDCA-3^+^ myDC was analyzed by flow cytometry. This graph depicts the percentage of each DC subpopulation in the live cell population. **(B)** BDCA-1^+^/BDCA-3^+^ myDC were incubated for 24h with supernatant from untreated 938-mel cells, supernatant from 938-mel cells treated with T-VEC (MOI 1) during 24h or supernatant from 938-mel cells treated with T-VEC (MOI 1) during 48 h. After 24 h, the phenotype of the DC was analyzed by flow cytometry. On the left y-axis (red symbols) the percentage of expression of each marker is depicted and on the right y-axis (blue symbols) the mean fluorescence intensity (MFI) is plotted. Each symbol represents the result of an independent experiment, and the line represents the median. **(C)** On the supernatants from the cultures described in **(B)**, cytokine content was analyzed using MesoScale Diagnostics assays. For IL-10 and TNFα, concentrations were normalized to the conditions with supernatant from untreated tumor cells (either 624-mel or 938-mel) because the absolute cytokine concentrations showed high variation among donors. For IFN-α-2a and IFN-λ1, the actual concentrations are plotted. The graphs depict mean values ± standard error of mean (SEM) and represent data from 6 independent experiments.

**Figure 3:**
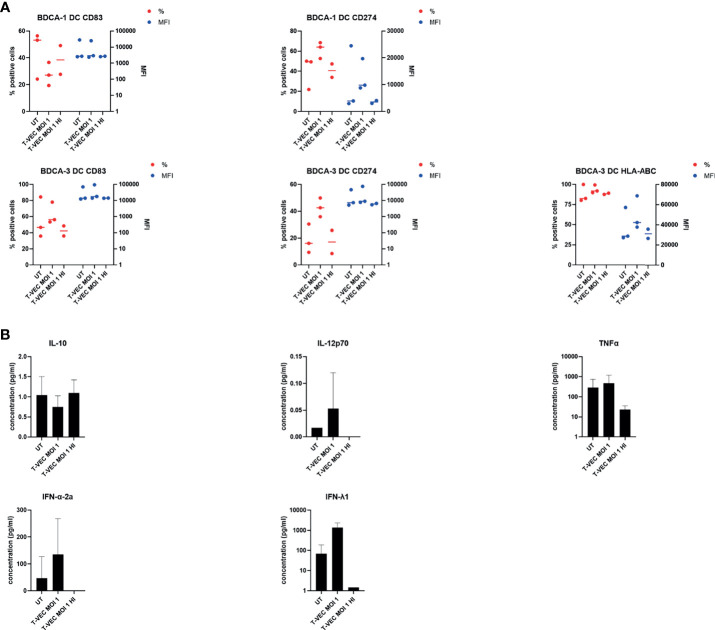
Effect of T-VEC on purified BDCA-1^+^/BDCA-3^+^ myDC. **(A)** BDCA-1^+^/BDCA-3^+^ myDC were incubated with T-VEC (MOI 1), heat-inactivated T-VEC (MOI 1) or left untreated. After 24h, the phenotype of the DC was analyzed by flow cytometry. On the left y-axis (red symbols) the percentage of expression of each marker is depicted and on the right y-axis (blue symbols) the mean fluorescence intensity (MFI) is plotted. Each symbol represents the result of an independent experiment and the line represents the median. **(B)** On the supernatants from the cultures described in **(B)**, cytokine content was analyzed using MesoScale Diagnostics assays. The graphs depict mean values ± SD and represent data from 3 independent experiments. For IL-12p70, some values were undetectable.

We cultured purified BDCA-1^+^/BDCA-3^+^ myDC in the presence of supernatant of T-VEC-treated melanoma cells and analyzed their phenotype and cytokine secretion pattern. **Figure 2A** shows the purity of the isolated BDCA-1^+^/BDCA-3^+^ myDC cell products. On average, the BDCA-1^+^/BDCA-3^+^ myDC cell product contained 68.86 ± 14.42% BDCA-1^+^ myDC and 6.71 ± 3.59% BDCA-3^+^ myDC, corresponding to a ratio of 10:1 as described before in the blood (7). Upon isolation, these BDCA-1^+^/BDCA-3^+^ myDC showed an immature phenotype (data not shown). After treatment with supernatant from T-VEC-infected tumor cells, we observed a trend towards an increase in expression of CD80, CD274 (PD-L1) and HLA-ABC on both the BDCA-1^+^ and BDCA-3^+^ myDC subsets, while levels of CD40, CD83 and CD86 remained constant. **Figure 2B** shows the data for CD80, CD274/PD-L1 and HLA-ABC using supernatant from 938-mel cells treated with T-VEC. Data for all markers and using another cell line 624-mel are shown in the **Supplementary Figure 1**. The supernatant from these myDC cultures was subsequently analyzed for its cytokine content and we observed a trend towards increased TNF-α, IFN-α-2a and IFN-λ1 concentrations upon culture in the presence of supernatant from T-VEC-treated melanoma cells, while IL-10 concentration remained unchanged (**Figure 2C**) and IL-12p70 was mainly undetectable (data not shown). These data show that the supernatant of T-VEC-treated melanoma cells contains inflammatory mediators that can induce a partial maturation in BDCA-1^+^/BDCA-3^+^ myDC. Since T-VEC might still be present in the supernatant from tumor cells, we also heat-inactivated the supernatant before addition to the BDCA-1^+^/BDCA-3^+^ myDC culture. We indeed observed a decreased cytokine production upon heat inactivation, but this effect was also observed in conditions without T-VEC (**Supplementary Figure 2**). To explore the immunostimulatory properties of T-VEC itself, we also analyzed the direct effect of T-VEC on BDCA-1^+^/BDCA-3^+^ myDC. T-VEC treatment did not exert an effect on the viability of BDCA-1^+^/BDCA-3^+^ myDC (data not shown), indicating it is not toxic. On BDCA-1^+^ DC, we observed trends towards a decrease in CD83 and an increase of CD274 expression upon treatment with T-VEC. On BDCA-3^+^ DC, we noted a trend to increased levels of HLA-ABC, CD83 and CD274 expression (**Figure 3A**). All other markers remained unchanged (**Supplementary Figure 3**). We also analyzed the cytokine secretion by BDCA-1^+^/BDCA-3^+^ myDC upon T-VEC treatment and noted an upregulation of IL-12p70, IFN-α-2a and IFN-λ1 (**Figure 3B**), although not statistically significant. These effects of T-VEC were abolished by heat inactivation before addition to the myDC cultures. These data show that the observed partial maturation effect of supernatant from T-VEC-treated melanoma cells is not completely mirrored by the effect of T-VEC itself. Moreover, since both T-VEC and supernatant from T-VEC-treated melanoma cells only show a trend towards a partial maturation of BDCA-1^+^/BDCA-3^+^ myDC, when compared to the complete maturation induced by the positive control (R848 + poly(I:C)) (data not shown), these data suggest that the potency of BDCA-1^+^/BDCA-3^+^ myDC treated with supernatant from T-VEC-treated melanoma cells could be enhanced further by the addition of a maturation stimulus.

### T-VEC-Treated Melanoma Cells Are Efficiently Taken up by BDCA-1^+^/BDCA-3^+^ myDC

**Figure 4:**
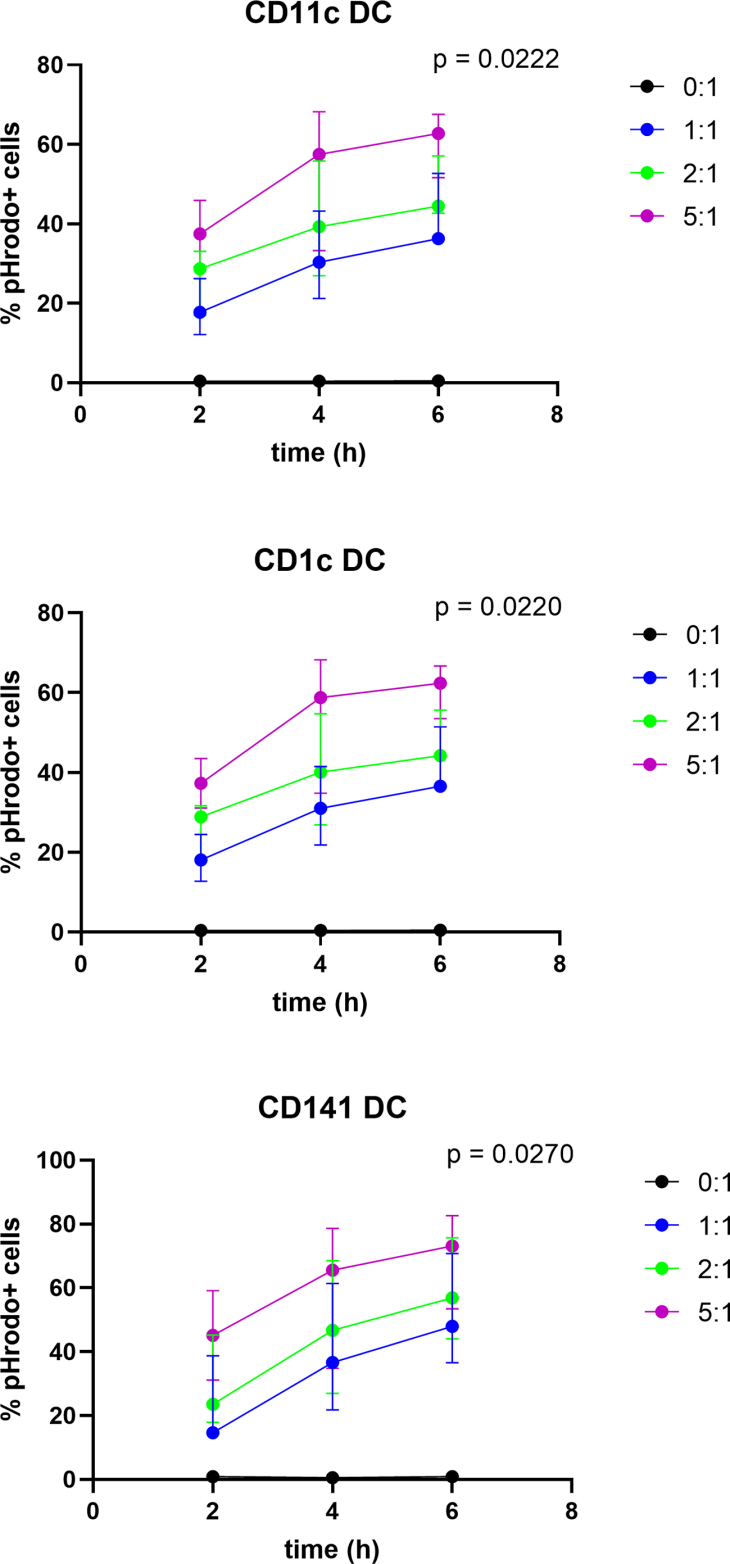
Uptake of T-VEC-treated dying melanoma cells by BDCA-1^+^/BDCA-3^+^ myDC. 938-mel cells were treated with T-VEC at a MOI of 1 for 24h. Then, cells were harvested, counted and labeled with the pHrodo™ Deep Red Mammalian and Bacterial Cell Labeling Kit. After labeling, labeled T-VEC-treated dying 938-mel cells were put in co-culture with purified BDCA-1^+^/BDCA-3^+^ myDC at the indicated ratios for 2, 4 or 6h. After co-culture for the indicated time points, cells were harvested and analyzed by flow cytometry. The different DC populations were gated and the percentage of pHrodo™-positive cells was determined. Each panel shows the uptake by the indicated DC population. Data are represented as mean ± SD from 3 independent experiments.

In order to investigate whether dying tumor cells, due to T-VEC treatment are taken up by BDCA-1^+^/BDCA-3^+^ myDC, we labeled the tumor cells with a pH-sensitive dye (pHrodo™ dye) before co-culturing the cells with BDCA-1^+^/BDCA-3^+^ myDC. The fluorochrome only becomes fluorescent in an acidic environment, so when the labeled cells are engulfed by DC and enter the acidic phagosome, the fluorescent signal becomes detectable. We co-cultured pHrodo™-labeled, T-VEC-treated 938-mel cells with BDCA-1^+^/BDCA-3^+^ myDC at different ratios (tumor cell:DC of 0:1, 1:1, 2:1 and 5:1) and during different incubation times (2-4-6h). Uptake of dying cells was assessed by flow cytometry. As shown in **Figure 4**, for each DC population (CD11c^+^ DC, CD1c^+^ DC and CD141^+^ DC), we noted an increased uptake over time as well as a higher uptake upon higher T:DC ratios. There was a trend towards a higher uptake by CD141^+^ DC compared to CD1c^+^ DC or CD11c^+^ total DC population, but this was not statistically significant. These data show that BDCA-1^+^/BDCA-3^+^ myDC are capable of engulfing cellular material from dying tumor cells.

### BDCA-1^+^/BDCA-3^+^ myDC Co-Cultured With Dying T-VEC-Treated Melanoma Cells Cross-Present Tumor Antigens to T Cells

**Figure 5:**
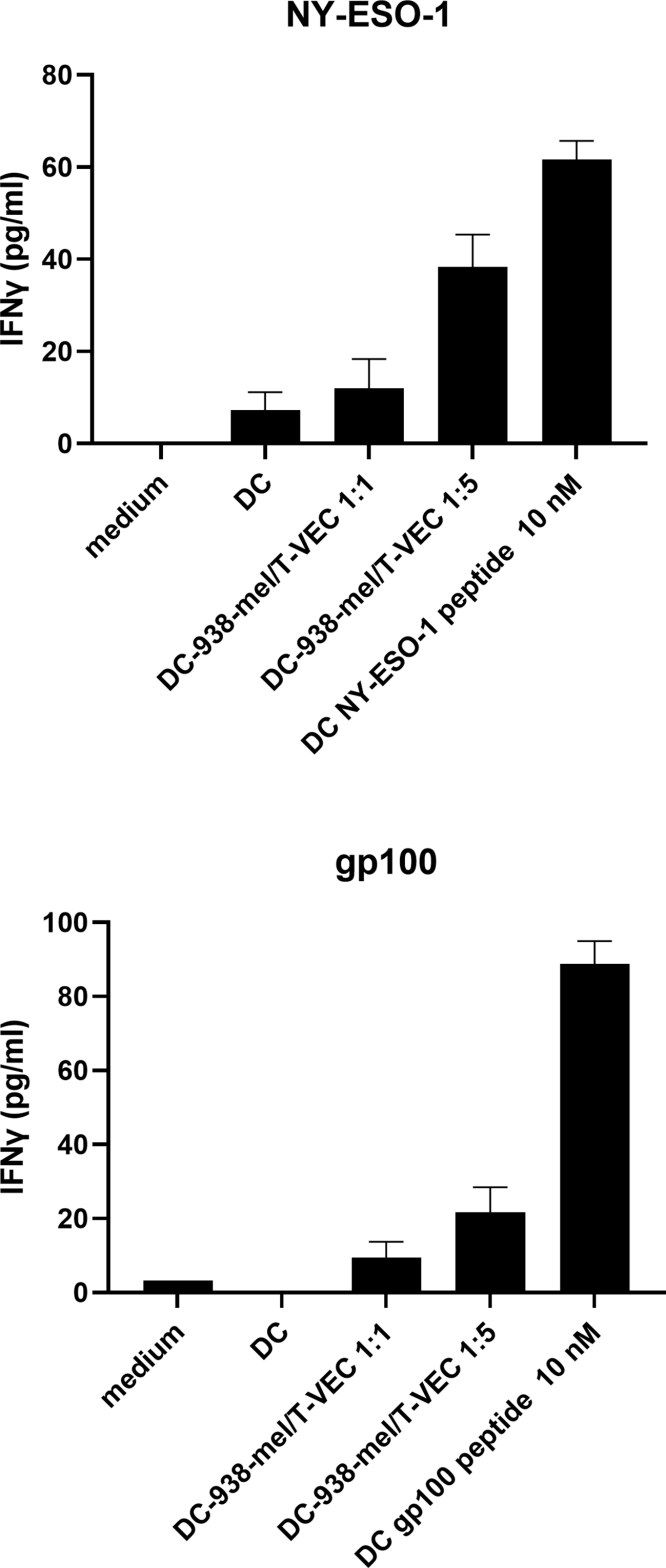
Antigen presentation by BDCA-1^+^/BDCA-3^+^ myDC that have taken up T-VEC-treated melanoma cells. 938-mel cells were treated with T-VEC at a MOI of 1 for 24h and subsequently co-cultured with BDCA-1^+^/BDCA-3^+^ myDC from an HLA-A2^+^ donor for 24h. After 24h, cells were harvested and counted and put into co-culture (1:1) with autologous T cells that were electroporated with mRNA encoding for the α- and β-chain of the TCR for NY-ESO-1 (top panel) or gp100 (bottom panel). After overnight co-culture of DC and TCR-transfected T cells, the supernatant was harvested and analyzed for IFN-γ content by ELISA. Data points depict the mean ± SD of 3 replicate wells.

Next, we explored whether BDCA-1^+^/BDCA-3^+^ myDC that are co-cultured with T-VEC-treated dying melanoma cells are capable of processing tumor antigens from the melanoma cells for presentation to T cells. To this end, we used BDCA-1^+^/BDCA-3^+^ myDC and T cells from an HLA-A2-positive patient. The T cells were transfected with mRNA encoding the α- and β-chain from the NY-ESO-1 TCR or the gp100 TCR to serve as a source of NY-ESO-1-specific and gp100-specific T cells respectively. HLA-A2-positive BDCA-1^+^/BDCA-3^+^ myDC were co-cultured during 24h with T-VEC-treated 938-mel cells (NY-ESO-1^+^, gp100^+^, HLA-A2^-^) and subsequently, these DC were put in co-culture with the autologous NY-ESO-1-specific or gp100-specific T cells and after overnight incubation the production of IFN-γ was measured. **Figure 5** shows that BDCA-1^+^/BDCA-3^+^ myDC co-cultured with T-VEC-treated 938-mel cells indeed induce IFN-γ secretion from both the NY-ESO-1 TCR transfected T cells as well as from the gp100 TCR transfected T cells. The effect was highest using a DC:tumor cell ratio of 1:5, although it remained lower than DC pulsed with 10 nM peptide. Thus, BDCA-1^+^/BDCA-3^+^ myDC that have taken up T-VEC-treated dying tumor cells are capable of cross-presenting tumor antigens to tumor-specific T cells.

## Discussion

In this study, we investigated the interaction between T-VEC, an oncolytic HSV-1 virus, melanoma cells and naturally circulating myDC (BDCA-1^+^ and BDCA-3^+^). The mechanism of action of T-VEC exists of three phases (1): inducing a lytic cancer cell death with release of tumor-associated antigens and immune stimulatory molecules (“danger” signals) (2), attracting and activating immature DC through production of GM-CSF and (3) inducing an adaptive immune response against the cancer cells (19). We showed that the supernatant of T-VEC-treated melanoma cells can partially activate BDCA-1^+^/BDCA-3^+^ myDC and that these BDCA-1^+^/BDCA-3^+^ myDC can take up co-cultured T-VEC-treated melanoma cells. Finally, we were able to show that BDCA-1^+^/BDCA-3^+^ myDC that have taken up dying T-VEC-treated melanoma cells are able to cross-present tumor antigens to antigen-specific T cells. To our knowledge, these findings have not yet been described using BDCA-1^+^/BDCA-3^+^ myDC.

Considering the effect of T-VEC on melanoma cell lines, our data confirms the findings that were reported previously by others: T-VEC effectively kills melanoma cells and a higher MOI results in a more rapid decrease of cell growth (17, 20). Other oncolytic viruses, such as Newcastle Disease Virus (NDV) have also been shown to be able to induce immunogenic apoptosis in the HER-2 positive human breast cancer cell line SK-BR-3 (21). However, we are the first to show the kinetics associated with T-VEC induced cell death. This allows future clinical trials to better estimate the optimal timing for administration of co-injectables such as DC or adjuvants. Except for the MOI 1 condition which induces almost immediate cell death, it takes 25-48 hours for T-VEC to induce cell death in melanoma cells.

Subsequently, we investigated the effect of both T-VEC itself as well as the supernatant from melanoma cell lines treated with T-VEC on myDC. T-VEC itself was not toxic for BDCA-1^+^/BDCA-3^+^ myDC. To investigate whether treatment of melanoma cells with T-VEC could result in the release of potent stimuli that could activate the injected DC, we *in vitro* treated BDCA-1^+^/BDCA-3^+^ myDC with supernatant from melanoma cells treated with T-VEC. We first focused on ‘phenotypic maturation’ by looking at the expression of classical maturation markers necessary for the activation of T cells such as CD40, CD80, CD86 and upregulation of HLA-ABC molecules. Next to this, we studied the release of cytokines that play a role in the anti-tumor immune response and have been shown to be produced by myDC. IL-10 is an immunosuppressive cytokine produced by a tolerogenic DC subset (22). IL-12 (with its functionally active heterodimer IL-12p70) promotes T cell differentiation into a Th1 effector type (T helper or cytotoxic T cell) (23). TNF-α is a pleiotropic cytokine with powerful immune stimulating functions. IFN-α-2a (type I interferon) and IFN-λ1 (type III interferon) are both induced in response to viral infections but also play a role in anti-tumor immunity. IFN-α-2a induces apoptosis in tumor cells and promotes NK and T-cell priming (24). IFN-λ has been discovered more recently and plays a role in triggering anti-tumor NK and T cells (25–29). Recently, it was shown that IFN-λ can be produced by BDCA-3^+^ DC (30). It has been shown previously by Bai et al. that NDV infected MCF-7 cells have beneficial effects on the antigen presentation capacity of breast cancer patient derived dendritic cells demonstrated by an upregulation of CD40, CD80, CD83, CD86 and MHC class II (HLA-DR). Within these co-cultures, increased levels of IFN-α, IL-12 and IL-15 could be detected (31, 32). More recently, it was shown that NDV infected SK-BR-3 cells are able to mature monocyte derived dendritic cells as demonstrated by the upregulation of CD40, CD80, MHC class I and II, increased levels of several cytokines (IFN-α, IL-6, TNF-α, IL-12) and chemokines (MIP-1α, RANTES, IP-10) (21). Within this context, we observed a trend to increased TNF-α, IFN-α-2a and IFN-λ1 secretion as well as an increased expression of CD80, PD-L1 and HLA-ABC by the BDCA-1^+^/BDCA-3^+^ myDC after treatment with supernatant from T-VEC treated melanoma cells. Heat inactivation of the tumor cell supernatant before addition to myDC resulted in decreased cytokine production, but also in conditions without T-VEC. To rule out that part of this effect might be related to the presence of T-VEC itself, we also tested the effect of T-VEC alone, which also resulted in a slight increase of IFN-α-2a and IFN-λ1 production. However, in response to T-VEC alone we also detected the production of IL-12p70 which was not seen when using supernatant. Since we only noted a partial maturation of BDCA-1^+^/BDCA-3^+^ myDC, our data suggest that the additional co-injection of an adjuvant capable of maturing these myDC might even improve the functionality of the myDC. In our hands, heat-inactivated T-VEC was unable to induce this partial DC maturation. This might indicate that either T-VEC needs to infect/enter DC to induce its maturing effect or alternatively, it could mean that important viral PAMPs are denatured by heat inactivation. The activation of PRR combined with cytokine signaling is needed to initiate a highly conserved cascade of genes regulated by transcription factors within DC, critical to control viral infections (33). It is currently not completely elucidated which PRR are involved in sensing of T-VEC, but for HSV-1, the virus from which T-VEC has been derived, it has been shown that multiple viral components can be recognized by different PRR (34). Viral proteins are recognized by TLR2 and herpes virus entry mediator (HVEM). Upon entry into the cell, viral DNA is sensed by TLR9 in endosomes, cyclic guanosine monophosphate-adenosine monophosphate synthase (cGAS) in the cytoplasm, IFNγ inducible protein 16 (IFI16) mostly in the nucleus, and DNA dependent activator of IFN-regulatory factors (DAI). During replication, double stranded RNA is sensed by TLR3 in endosomes, melanoma differentiation-associated protein 5 (MDA5), RIG-I, and protein kinase RNA-activated (PKR) in the cytoplasm. Both HSV-1 and T-VEC have been shown to activate STING *via* the cGAS sensor (17, 35). It remains to be established which other PRR are involved in recognition of T-VEC.

It has already been shown that phagocytes such as macrophages or DC are able to take up material from dying tumor cells (21, 31, 32, 36–39). However, we are not aware that this has been shown before using natural BDCA-1^+^/BDCA-3^+^ myDC. We were able to show that each myDC subtype (both the complete CD11c^+^ population, as well as the BDCA-1^+^ and BDCA-3^+^ subsets separately) was capable to engulf melanoma cells with a trend towards higher uptake by BDCA-3^+^ DC which is expected as these cells are specialized in cross-presentation of (tumor) antigens (40).

It has been shown previously that monocyte-derived DC loaded with tumor antigens, derived from whole tumor cell lysates, are efficacious antigen-presenting cells able to initiate a T cell response against malignant glioma tumor cells showed by the upregulation of CD25 on CD8^+^ T cells and by the generation of cytotoxicity against the target cells (39). Moreover, it has also been suggested that DC loaded with NDV-derived viral oncolysates might stimulate more potent T cell responses compared to DC pulsed with tumor lysate without NDV (31, 41). Hence, since myDC are known for their unique cross-presentation capabilities (4, 5), we were interested to see if the activated and loaded DC were subsequently able to cross-present melanoma-specific tumor antigens to antigen-specific T cells. To answer this question, we electroporated T cells with the T cell receptor for NY-ESO-1 and gp100, two melanoma-associated tumor antigens. We then co-cultured BDCA-1^+^/BDCA-3^+^ myDC with T-VEC-treated melanoma cells for 24 hours, followed by co-culture with the electroporated T cells. As we have demonstrated, BDCA-1^+^/BDCA-3^+^ myDC co-cultured with T-VEC-treated melanoma cells indeed induced secretion of IFN-γ by T cells, indicating that the melanoma antigens were indeed cross-presented, although at low levels compared to peptide-pulsed myDC. To our knowledge, we are the first to show that BDCA-1^+^/BDCA-3^+^ myDC are capable of cross-presenting tumor-associated antigens released by T-VEC treated melanoma cells to antigen-specific T cells.

At present, a phase I clinical trial is being conducted where T-VEC and myDC are co-injected intratumorally (ClinicalTrials.gov Identifier: NCT03747744). Early results from this trial indicate that durable tumor responses can be obtained in patients with immune checkpoint inhibitor refractory melanoma (42).

For future translation of this type of clinical trials involving academic cell therapy production to a larger patient population, several practical and logistic hurdles remain. There is an important need for exchange of best practices regarding transportation (to/from a GMP facility), cryopreservation, manipulation and manufacturing of cell therapy products. However, as the recent development and FDA approval of CAR-T cell therapies such as Yescarta^®^ and Kymriah^®^ have demonstrated it is not impossible to scale cell-therapy development and make it accessible to more patients on a global scale (43).

In the future, more research is needed regarding the combination of intratumoral treatment modalities such as T-VEC and (intratumorally injected) myDC. It is yet unclear how important the role of the induced cell lysis by T-VEC is *in vivo*. Next to this, we know that more potent maturation factors exist for these DC subtypes, but these remain as yet unavailable for clinical (intratumoral) use. Both clinical and pre-clinical validation of promising combinations is warranted for future combination therapies involving myDC.

In conclusion, we have shown that the combination of T-VEC with BDCA-1^+^/BDCA-3^+^ myDC is complementary and able to *ex vivo* induce an immune response against melanoma cells. We have shown that melanoma cells are killed after infection with T-VEC, resulting in the release of tumor antigens and partial maturation of BDCA-1^+^/BDCA-3^+^ myDC. These myDC are able to take up melanoma antigens, process these antigens and subsequently present these antigens to antigen-specific T cells. These experiments show that the combination of intratumoral injection of myDC and T-VEC are complementary treatment modalities that could lead to an adaptive immune response against melanoma and should be further explored in the clinical setting.

## Data Availability Statement

The raw data supporting the conclusions of this article will be made available by the authors, without undue reservation.

## Ethics Statement

The studies involving human participants were reviewed and approved by Ethics committee of the UZ Brussel. The patients/participants provided their written informed consent to participate in this study.

## Author Contributions

BN, KB, and JA conceptualized the design of the study. JT, JDM, ID, JKS, LF, KB and ST were responsible for the experimental design. JT, IVR and ST acquired the data. JT and ST analyzed the data and drafted the manuscript. All authors critically revised the manuscript and approved the submitted version.

## Funding

This study is funded by “Kom op tegen Kanker (Stand up to Cancer), the Flemish cancer society”.

## Conflict of Interest

BN and JS received non-financial support from Amgen.

The remaining authors declare that the research was conducted in the absence of any commercial or financial relationships that could be construed as a potential conflict of interest.

## Publisher’s Note

All claims expressed in this article are solely those of the authors and do not necessarily represent those of their affiliated organizations, or those of the publisher, the editors and the reviewers. Any product that may be evaluated in this article, or claim that may be made by its manufacturer, is not guaranteed or endorsed by the publisher.

## Supplementary Material

Supplementary Figure 1 Effect of supernatant from T-VEC-treated melanoma cells on BDCA-1+/BDCA-3+ myDC. BDCA-1+/BDCA-3+ myDC were incubated for 24h with supernatant from untreated 624-mel cells, supernatant from 624-mel cells treated with T-VEC (MOI 1) during 24h, supernatant from 624-mel cells treated with supernatant from 624-mel cells treated with T-VEC (MOI 1) during 48h, supernatant from untreated 938-mel cells, supernatant from 938-mel cells treated with T-VEC (MOI 1) during 24h or supernatant from 938-mel cells treated with T-VEC (MOI 1) during 48h. After 24h, the phenotype of the DC was analyzed by flow cytometry. **(A)** Results gated on BDCA-1+ DC. **(B)** Results gated on BDCA-3+ DC. On the left y-axis (red symbols) the percentage of expression of each marker is depicted and on the right y-axis (blue symbols) the mean fluorescence intensity (MFI) is plotted. Each symbol represents the result of an independent experiment, and the line represents the median.

Supplementary Figure 2 Effect of heat inactivation of supernatant from T-VEC-treated melanoma cells on BDCA-1+/BDCA-3+ myDC cytokine secretion. BDCA-1+/BDCA-3+ myDC were incubated for 24h with supernatant from untreated 938-mel cells, supernatant from 938-mel cells treated with T-VEC (MOI 1) during 24h or supernatant from 938-mel cells treated with T-VEC (MOI 1) during 48h. The supernatant was either added as such or after a heat-inactivation step. After 24h, the supernatant from the cultures was harvested and cytokine content was analyzed using MesoScale Diagnostics assays. The cytokine concentrations were normalized to the condition where the cells were incubated with the supernatant of untreated tumor cells (both for 624-mel and 938-mel). Each symbol represents the result of an independent experiment, and the bars indicate the mean ± SD.

Supplementary Figure 3 Effect of T-VEC on BDCA-1+/BDCA-3+ myDC. BDCA-1+/BDCA-3+ myDC were incubated with T-VEC (MOI 1), heat-inactivated T-VEC (MOI 1) or left untreated. After 24h the phenotype of the DC was analyzed by flow cytometry. **(A)** Results gated on BDCA-1+ DC. **(B)** Results gated on BDCA-3+ DC. On the left y-axis (red symbols) the percentage of expression of each marker is depicted and on the right y-axis (blue symbols) the mean fluorescence intensity (MFI) is plotted. Each symbol represents the result of an independent experiment, and the line represents the median.
