# Supplementary figures and images for: Unraveling the Effects of a Talimogene Laherparepvec (T-VEC)-Induced Tumor Oncolysate on Myeloid Dendritic Cells
Source: Front Immunol. 2021 Oct 28;12:733506. doi: 10.3389/fimmu.2021.733506 (PMC8581672; doi:10.3389/fimmu.2021.733506)

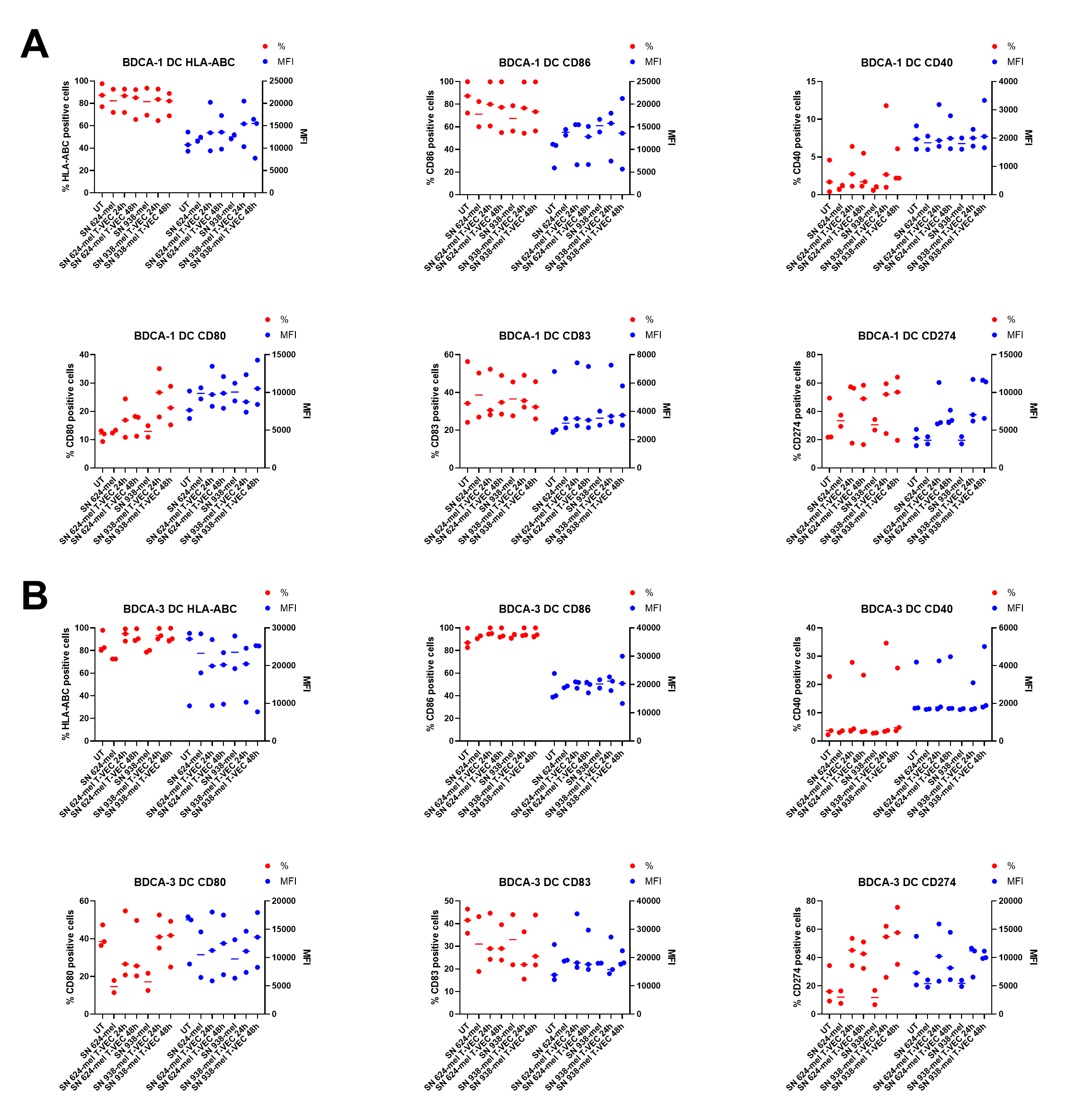

Supplement: Supplementary Figure 1 — Effect of supernatant from T-VEC-treated melanoma cells on BDCA-1+/BDCA-3+ myDC. BDCA-1+/BDCA-3+ myDC were incubated for 24h with supernatant from untreated 624-mel cells, supernatant from 624-mel cells treated with T-VEC (MOI 1) during 24h, supernatant from 624-mel cells treated with supernatant from 624-mel cells treated with T-VEC (MOI 1) during 48h, supernatant from untreated 938-mel cells, supernatant from 938-mel cells treated with T-VEC (MOI 1) during 24h or supernatant from 938-mel cells treated with T-VEC (MOI 1) during 48h. After 24h, the phenotype of the DC was analyzed by flow cytometry. (A) Results gated on BDCA-1+ DC. (B) Results gated on BDCA-3+ DC. On the left y-axis (red symbols) the percentage of expression of each marker is depicted and on the right y-axis (blue symbols) the mean fluorescence intensity (MFI) is plotted. Each symbol represents the result of an independent experiment, and the line represents the median. [file Image_1.tif]

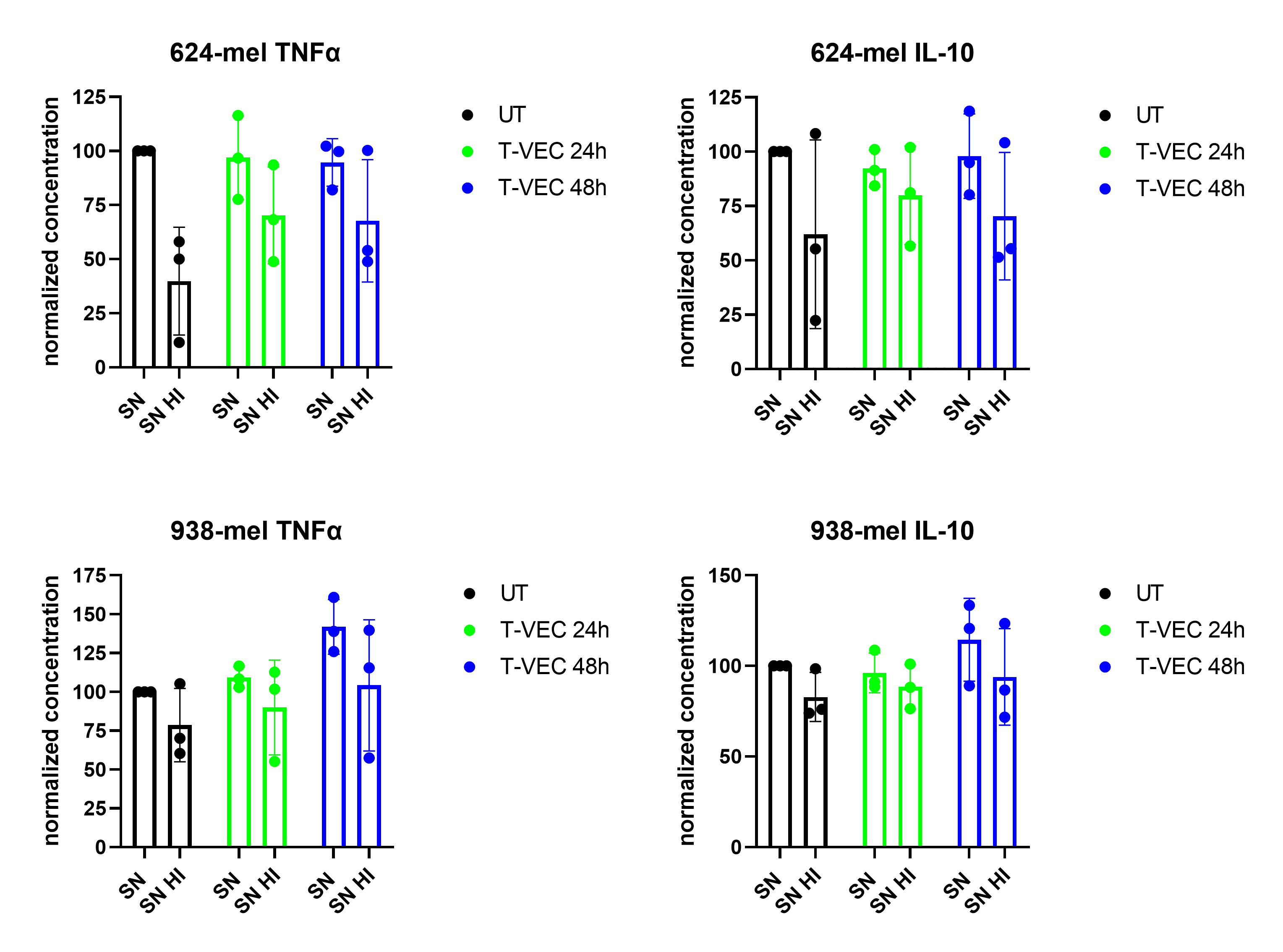

Supplement: Supplementary Figure 2 — Effect of heat inactivation of supernatant from T-VEC-treated melanoma cells on BDCA-1+/BDCA-3+ myDC cytokine secretion. BDCA-1+/BDCA-3+ myDC were incubated for 24h with supernatant from untreated 938-mel cells, supernatant from 938-mel cells treated with T-VEC (MOI 1) during 24h or supernatant from 938-mel cells treated with T-VEC (MOI 1) during 48h. The supernatant was either added as such or after a heat-inactivation step. After 24h, the supernatant from the cultures was harvested and cytokine content was analyzed using MesoScale Diagnostics assays. The cytokine concentrations were normalized to the condition where the cells were incubated with the supernatant of untreated tumor cells (both for 624-mel and 938-mel). Each symbol represents the result of an independent experiment, and the bars indicate the mean ± SD. [file Image_2.tif]

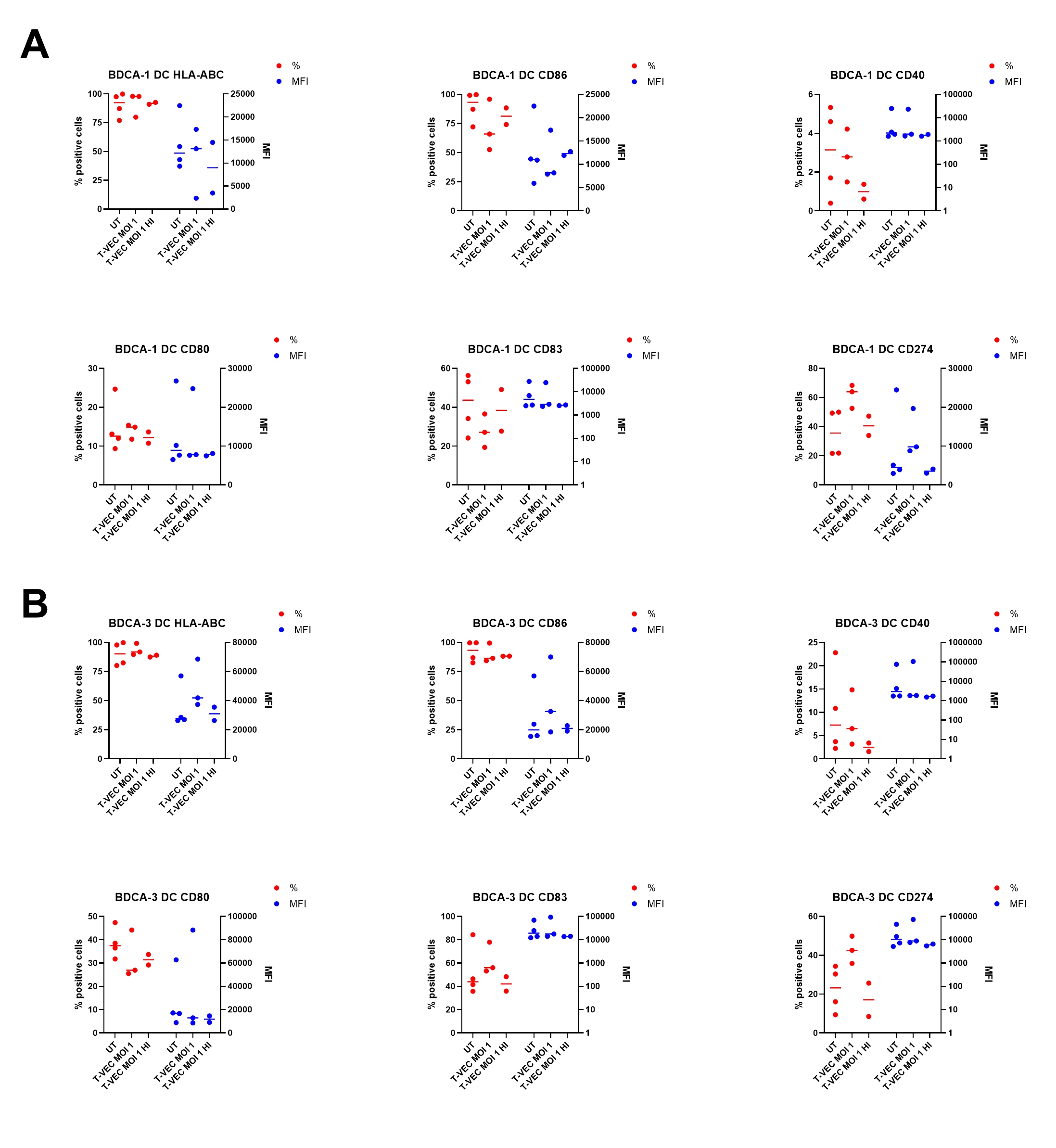

Supplement: Supplementary Figure 3 — Effect of T-VEC on BDCA-1+/BDCA-3+ myDC. BDCA-1+/BDCA-3+ myDC were incubated with T-VEC (MOI 1), heat-inactivated T-VEC (MOI 1) or left untreated. After 24h the phenotype of the DC was analyzed by flow cytometry. (A) Results gated on BDCA-1+ DC. (B) Results gated on BDCA-3+ DC. On the left y-axis (red symbols) the percentage of expression of each marker is depicted and on the right y-axis (blue symbols) the mean fluorescence intensity (MFI) is plotted. Each symbol represents the result of an independent experiment, and the line represents the median. [file Image_3.tif]
